# Multi-Epitope-Based Vaccines for Colon Cancer Treatment and Prevention

**DOI:** 10.3389/fimmu.2021.729809

**Published:** 2021-08-30

**Authors:** Lauren R. Corulli, Denise L. Cecil, Ekram Gad, Marlese Koehnlein, Andrew L. Coveler, Jennifer S. Childs, Ronald A. Lubet, Mary L. Disis

**Affiliations:** ^1^University of Washington (UW) Medicine, Cancer Vaccine Institute, University of Washington, Seattle, WA, United States; ^2^Division of Cancer Prevention, National Cancer Institute, Bethesda, MD, United States

**Keywords:** immunotherapy, colon cancer, CDC25B, COX2, IFN-gamma

## Abstract

**Background:**

Overexpression of nonmutated proteins involved in oncogenesis is a mechanism by which such proteins become immunogenic. We questioned whether overexpressed colorectal cancer associated proteins found at higher incidence and associated with poor prognosis could be effective vaccine antigens. We explored whether vaccines targeting these proteins could inhibit the development of intestinal tumors in the azoxymethane (AOM)-induced colon model and APC Min mice.

**Methods:**

Humoral immunity was evaluated by ELISA. Web-based algorithms identified putative Class II binding epitopes of the antigens. Peptide and protein specific T-cells were identified from human peripheral blood mononuclear cells using IFN-gamma ELISPOT. Peptides highly homologous between mouse and man were formulated into vaccines and tested for immunogenicity in mice and *in vivo* tumor challenge. Mice treated with AOM and APC Min transgenic mice were vaccinated and monitored for tumors.

**Results:**

Serum IgG for CDC25B, COX2, RCAS1, and FASCIN1 was significantly elevated in colorectal cancer patient sera compared to volunteers (CDC25B p=0.002, COX-2 p=0.001, FASCIN1 and RCAS1 p<0.0001). Epitopes predicted to bind to human class II MHC were identified for each protein and T-cells specific for both the peptides and corresponding recombinant protein were generated from human lymphocytes validating these proteins as human antigens. Some peptides were highly homologous between mouse and humans and after immunization, mice developed both peptide and protein specific IFN-γ-secreting cell responses to CDC25B, COX2 and RCAS1, but not FASCIN1. FVB/nJ mice immunized with CDC25B or COX2 peptides showed significant inhibition of growth of the syngeneic MC38 tumor compared to control (p<0.0001). RCAS1 peptide vaccination showed no anti-tumor effect. In the prophylactic setting, after immunization with CDC25B or COX2 peptides mice treated with AOM developed significantly fewer tumors as compared to controls (p<0.0002) with 50% of mice remaining tumor free in each antigen group. APC Min mice immunized with CDC25B or COX2 peptides developed fewer small bowel tumors as compared to controls (p=0.01 and p=0.02 respectively).

**Conclusions:**

Immunization with CDC25B and COX2 epitopes consistently suppressed tumor development in each model evaluated. These data lay the foundation for the development of multi-antigen vaccines for the treatment and prevention of colorectal cancer.

## Introduction

Active immunization as a colorectal cancer treatment and prevention strategy offers several advantages to classic drug-based approaches. Vaccines are administered over a short period of time without the need for daily dosing. Vaccines that induce tumor trafficking Type I T-cells, including CD8^+^ T-cells, could be used in combination with immune checkpoint inhibitor therapy to potentially increase clinical responses. Immunologic memory is generated ensuring an adaptive cellular immune response poised to eliminate aberrant cells at the time they arise for prevention of disease recurrence or even primary prevention. Once primed by a vaccine, T-memory cells, are active for years and can be boosted periodically with further vaccinations. In addition, vaccines have been shown to be non-toxic (1).

There have been numerous clinical studies immunizing patients against proteins expressed in the colon with limited to no adverse events. Indeed, a single antigen vaccine targeting MUC-1 has progressed to clinical evaluation in patients with previous high-risk adenomas as a first attempt in colorectal cancer immuno-prevention (2). The vaccine was immunogenic, generating high levels of MUC-1 specific antibodies, and was safe with few reported adverse effects. However, studies in transgenic mouse models have shown that multi-antigen vaccines are significantly more effective in inhibiting the progression of pre-invasive to invasive cancer and preventing clinical disease than single antigen vaccines alone (3).

Vaccines have had remarkable success in preventing cancers of viral origin such as hepatitis B and human papillomavirus, in part because the vaccines targeted proteins that drive oncogenesis. That success is extending to cancer treatment. A human papillomavirus vaccine as a treatment partner with an immune checkpoint inhibitor resulted in increased clinical responses as compared to what would be expected with an immune checkpoint inhibitor alone (4). In order to extrapolate similar success to the prevention of colorectal cancer, we need well-defined, biologically relevant, immunogenic proteins that are important to maintaining the malignant phenotype and can be targeted in a multi-antigen vaccine. To identify colorectal cancer associated antigens, we focused on two major characteristics of the protein candidates. The first was overexpression of the protein in cancer as compared to normal tissues. Aberrant overexpression of a protein in the malignant state is a major mechanism by which that protein becomes immunogenic (5). The second characteristic was the importance of the protein in colorectal cancer growth. This importance could be assessed by the expression of the protein negatively influencing prognosis or that protein having an essential biologic function in colorectal cancer pathogenesis. We identified four candidates based on these and other characteristics described below; CDC25B, COX2, FASCIN1 and RCAS1. CDC25B is overexpressed in over 40% of colorectal cancers and in multivariate analysis is an independent predictor of poor prognosis (risk ratio for death 3.7 as compared to non-expressers) (6). The CDC25B protein phosphatase regulates the cell cycle by activating cyclin-dependent protein kinases (7). In pre-clinical models CDC25 is essential to proliferation of intestinal epithelial stem cells and overexpression could be an early alteration in colorectal cancer pathogenesis (7). COX-2 is overexpressed in a majority of colon cancers and overexpression is also linked with a poor prognosis (8). In a study of over 660 colon cancer cases, COX-2-positive tumors were associated with an increased cancer-specific mortality (multivariate HR, 2.12; 95% CI, 1.23-3.65) (8). In addition, over 50% of adenomatous polyps overexpress the COX-2 protein as compared to adjacent normal tissues (9). Overexpression of COX-2 correlated with increasing adenoma size and an increasing degree of epithelial dysplasia (9). FASCIN1 and RCAS1 have been reported to be overexpressed in 26% of colon adenocarcinomas and 100% of metastatic lymph nodes from colorectal cancer patients respectively (10, 11). Strong diffuse expression of FASCIN1 is associated with a worse prognosis as compared to patients with FASCIN1 negative tumors, p=0.023 (10). RCAS1 expression imparts a poorer prognosis for colon cancer patients and protein expression has been reported to induce apoptosis in tumor infiltrating lymphocytes (12).

Here we describe the antigenicity of these colorectal cancer associated proteins, identify T-cell epitopes suitable for inclusion in a vaccine, and demonstrate how a vaccine with two of the antigens, CDC25B and COX2, consistently reduced tumor development in two mouse models.

## Material and Methods

### Human Subjects

The colorectal cancer patients (n=50) ranged in age from 31-89 (median age 61), and 50% were female. Stage I (12%), stage II (24%), stage III (24%) and stage V (40%) patient sera were evaluated (purchased from Innovative Research). Volunteer donors (n=50) ranged in age from 23-84 (median age 52.7), and 32% were female (Puget Sound Blood Bank). All donors met criteria for blood donation. Sera were aliquoted and stored at -80°C until use. For IFN-γ-secreting-cell studies, peripheral blood mononuclear cells (PBMC) were obtained from either a single blood draw of 10 patients with colorectal cancer in remission and at least 30 days from the end of chemotherapy or by leukapheresis from 10 volunteer donors, all after informed consent. Cells were cryopreserved as previously described (13).

### Identification of Antigens

We performed a literature search in PubMed with search terms “protein overexpression” and “poor prognosis” and “colorectal cancer”, using the following criteria to identify candidate vaccine antigens from the resultant proteins: (1) a greater than 20% incidence of overexpression in colon cancer, (2) a predictor of poor prognosis, and/or (3) a predictor of early disease recurrence, and (4) known biologic function in colon cancer pathogenesis. Four candidate antigens were selected for evaluation: CDC25B, COX2, FASCIN1 and RCAS1.

### Evaluation of Antigen-Specific Humoral Immunity

Indirect ELISA was performed as previously described with the following modifications: recombinant proteins CDC25B, COX2, FASCIN1, and RCAS1 (Novus Biologicals) were diluted with carbonate buffer to a concentration of 1 µg/ml (14). Data are presented as antigen specific IgG in µg/ml. A sample was defined as positive when the serum IgG value was greater than the mean and two standard deviations of the control sera (n=50) evaluated for each protein. The cutoff was determined at 0.51 µg/ml for CDC25B, 0.63 µg/ml for COX2, 1.44 µg/ml for FASCIN1, and 2.12 µg/ml for RCAS1. ELISA results were validated by Western Blotting on individual strips of nitrocellulose as previously described with a sensitivity and specificity, respectively of 83% and 83% for CDC25B, 100% and 100% for COX2, 100% and 100% for FASCIN1, and 100% and 100% for RCAS1 (**Supplementary Figure 1**) (14).

### Analysis of Peptide and Protein-Specific T-Cell Responses

Peptides predicted to promiscuously bind human MHC II were selected as previously described (15). Briefly, a combined scoring system using three widely available algorithms for predicting class II binding was used for the 15 most common MHC class II alleles (DRB1*0101, DRB1*1501, DRB1*0301, DRB1*0401, DRB1*0404, DRB1*0405, DRB1*0701, DRB1*0802, DRB1*0901, DRB1*1101, DRB1*1201, DRB1*1302, DRB3*0101, DRB4*0101, and DRB5*0101). For each available MHC class II allele, 20 peptide sequences (15-20mer) were initially selected solely based on the rank order of the predicted binding affinity. Scores (S) for each amino acid were summed up across the multiple MHC class II alleles from all three algorithms. The number (N) of MHC class II alleles for which each amino acid was predicted to have high affinity binding was counted. A rank score for each amino acid was defined as S x N. The following three algorithms were used for prediction of class II peptides derived from each protein sequence: SYFPEITHI (Institute for Cell Biology, Heidelberg, Germany), Propred (Institute of Microbial Technology, Chandigarh, India) and Rankpep (Harvard, Boston, MA).

Peptides chosen for evaluation encompassed at least 25% of the predicted high affinity binding regions of each protein and shared >75% homology with mouse (**Supplementary Table 1**). The peptides were synthesized and purified by high-performance liquid chromatography (>95% purity, Genemed Synthesis Inc). Human PBMC were evaluated by ELISPOT for antigen-specific IFN-gamma (γ) as previously described (16, 17). The ELISPOT used 10 µg/ml each of experimental peptide and the negative control peptide HIVp52 as well as 1 µg/ml of the corresponding recombinant protein (Novus Biologicals) and the positive controls of peptide pools for Cytomegalovirus, Epstein-Barr Virus and Influenza Virus (CEF; Anaspec) or tetanus toxoid (List Biological Laboratories). Human peptide T-cell lines were generated as previously described from volunteer donors demonstrating significant IFN-γ responses to the selected epitopes (18). The T-cell lines were assayed *via* IFN-γ ELISPOT using 1 µg/ml experimental recombinant protein (Novus Biologicals) and negative control recombinant protein Cyclin B (US Biologicals) and 10 µg/ml each of experimental peptides and the negative control peptide TRIP13-p104. Data for ELISPOTS are reported as corrected spots/well (cSPW) which is the mean number of spots for each experimental antigen minus the mean number of spots detected in no antigen control wells ± SEM.

Mouse splenic cells were evaluated by ELISPOT for antigen-specific IFN-γ secretion as published, except for the following modifications; splenic cells were incubated with antigens for 72 hours and spots were developed with the AEC substrate kit (BD Biosciences) (17). Data are reported as cSPW for individual antigens.

### Animal Models and Cell Line

Animal care and use were in accordance with institutional guidelines. APC Min [Strain name: C57BL/6J-ApcMin/J] males, C57BL6/J females, and FVB/nJ mice were purchased from Jackson Laboratory and allowed to acclimate for one week before treatment. The offspring from mated APC Min males and C57BL6/J females were genotyped by PCR for the presence of the Min mutation using primers as follows: Wild-Type: 5’-GCCATCCCTTCACGTTAG-3’, Common: 5’-TTCCACTTTGGCATAAGGC-3’, Mutant: 5’-TCCTGAGAAAGACAGAAGTTA-3’. Both male and female mice testing positive for the Min mutation were included in the study and randomized into treatment groups. For the AOM model, FVB/nJ mice were treated with 10 mg/kg of AOM (Sigma-Aldrich) twice a week *via* intraperitoneal (ip) injection for six weeks (19). A power analysis determined that 4 to 8 mice/group would achieve a power of at least 93.8% to detect difference between groups. The mouse colon cancer cell line derived from an AOM induced tumor (kindly provided by Dr. David Threadgill), MC38, was validated by IDEXX testing.

### Vaccination and Assessment of Tumor Growth

FVB/nJ, C57BL6/J or APC Min offspring were immunized subcutaneously at 6 ± 2 weeks of age with 50 µg each peptide in a pool per antigen with equal volume of adjuvant, PBS alone, or adjuvant alone. Complete Freund’s Adjuvant was used in the first immunization, followed by Incomplete Freund’s Adjuvant (Sigma-Aldrich) for subsequent immunizations for a total of three immunizations two weeks apart. Peptide vaccine boosters were given at 15-16 and 21-22 weeks of age. AOM treatments began two weeks after the final vaccine and mice were sacrificed at 26 weeks of age. APC Min mice were sacrificed at 16 ± 2 weeks. The entire colon from the AOM model or entire small intestine from APC Min mice were removed at sacrifice and cut longitudinally with dissection scissors. Fecal matter was washed off then tissues were fixed in formalin for a minimum of 24 hours. Intestinal tracts were removed from formalin and tumors were counted under a Nikon SMZ645 microscope by the same operator for each experiment.

For the tumor implant model, 4x10^4^ MC38 cells were implanted subcutaneously in C57BL6/J mice 2 weeks after the final vaccine. Mice were monitored for tumor growth every 2-3 days using Vernier calipers as previously described (20). Mice were sacrificed upon reaching a cumulative tumor volume of 1200mm^3^ or if tumors developed ulcers. Depletion of CD4^+^ and CD8^+^ T-cells was performed as published (15). Briefly, 100 µg anti-CD8 or 250 µg anti-CD4 monoclonal antibodies (UCSF) were injected i.p for 3 consecutive days before the first vaccine. The treatment was repeated twice weekly until termination of the study. This regimen resulted in >95% CD4+ or CD8+ T-cell depletion. Tumor growth for all experiments is reported as mean (± SEM) tumor volume (mm^3^) (15).

### Immunohistochemistry

Immunohistochemistry was performed on colon tumors from the AOM model as previously described (20). Briefly, the fixed sections cut from frozen blocks were blocked with 10% goat serum (Vector Labs) for 1 hour at room temperature then incubated overnight with rat-anti-mouse CD8 (clone KT15; 1:100; AbD Serotec) or rat-anti-mouse CD4 (clone 4SM95; 5 µg/ml; eBioscience). After extensive washing, the slides were incubated with Alexa Fluor 488 goat-anti-rat (Abcam; 1:500) for 1 hour at room temperature. Cover slips were mounted with Prolong Gold antifade with DAPI (Life technologies). Positive cells and DAPI stained nuclei were counted in three random high-powered fields per slide and expressed as a mean percent of positive stained cells of total cell counted.

### Statistical Analysis

Model assumptions were checked using the Shapiro-Wilk normality test and by visual inspection of residual and fitted value plots. The unpaired, two-tailed Student’s t-test and ANOVA test was used to evaluate differences when normality was confirmed. When normality of the data was not confirmed, the non-parametric Kruskal-Wallis and Man-Whitney tests were used. Differences in tumor volume was determined by two-way ANOVA with a Dunnett post-test for multiple comparisons. A p value of <0.05 was considered significant (GraphPad Software, Prism v.8).

## Results

### CDC25B, COX2, FASCIN1, and RCAS1 Are Colon Cancer Antigens

**Figure 1:**
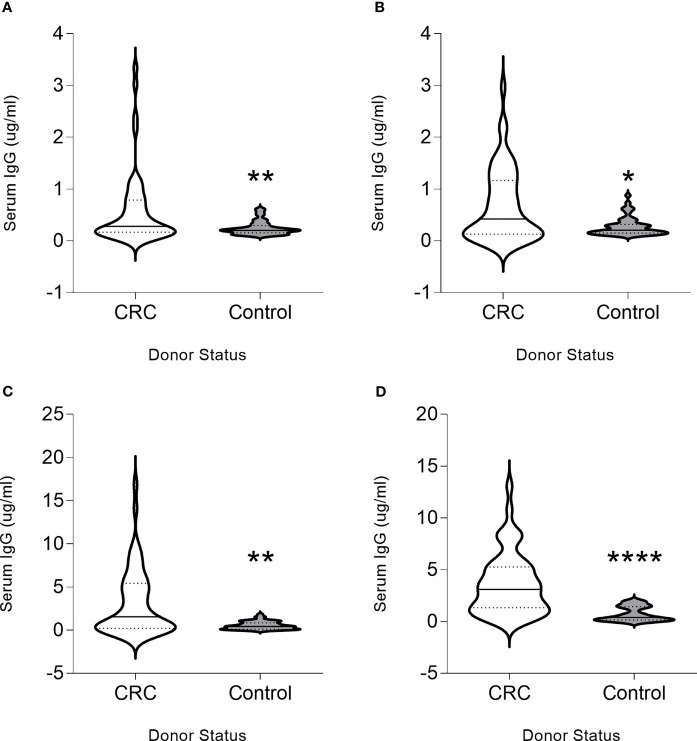
CDC25B, COX2, FASCIN1 and RCAS1 are colon cancer antigens. Serum IgG (µg/ml) from donors with colorectal cancer (CRC) or volunteer controls (control) for **(A)** CDC25B, **(B)** COX2, **(C)** FASCIN1 and **(D)** RCAS1 presented as violin plots, solid line at median and dotted lines at quartiles. *p < 0.05, **p < 0.01, ****p < 0.0001.

To determine whether the chosen proteins were immunogenic, we evaluated whether a humoral immune response could be detected. IgG antibodies were detected at higher magnitude in colon cancer patients as compared to controls for all proteins. Significantly higher levels of CDC25B-specific IgG antibodies were observed in patients with colorectal cancer (median, 0.28 µg/ml; range, 0.01-3.34 µg/ml) as compared to volunteer donors (median, 0.20 µg/ml; range, 0.1-0.61 µg/ml; p=0.041; **Figure 1A**). Thirty-six percent of colorectal cancer patients and eight percent of volunteer donors were seropositive for this antigen. Serum COX2 IgG was significantly elevated in the patients (median, 0.447 µg/ml; range, 0-2.97 µg/ml) compared to the control (median, 0.21 µg/ml; range, 0.10-0.87 µg/ml p=0.023; **Figure 1B**). Forty-two percent of colorectal cancer patients and six percent of volunteer donors were seropositive for this antigen. FASCIN1-specific IgG levels were greater in colorectal cancer sera (median, 1.55µg/ml; range, 0-16.88 µg/ml) compared to control donor sera (median, 0.42µg/ml; range; 0.01-1.81 µg/ml; p=0.008; **Figure 1C**). Fifty percent of colorectal cancer patients and six percent of volunteer donors were seropositive for this antigen. Greater RCAS1-specific serum IgG was detected in patients (median, 3.11µg/ml; range, 0.03-13.07) as compared to the control (median, 0.39µg/ml; range, 0.01-2.03 µg/ml; p<0.001; **Figure 1D**). Sixty-two percent of colorectal cancer patients and no volunteer donors were seropositive for this antigen. Increased levels of IgG was positively correlated with increased stage of colorectal cancer for FASCIN1 (p=0.04) and RCAS1 (p=0.008) and no correlation was associated with CDC25B or COX2. Only increased FASCIN1-specific IgG levels in colorectal cancer was negatively correlated with the age of the donor (p=0.006). Both CDC25B-specific and COX2-specific IgG levels were higher in males than females (p=0.009 and p=0.010, respectively) whereas no difference between males and females were observed for FASCIN1 and RCAS.

### IFN-γ-Secreting Cells Specific for Both Peptide and Protein Antigens Can Be Identified in the Peripheral Blood of Colorectal Cancer Patients

**Figure 2:**
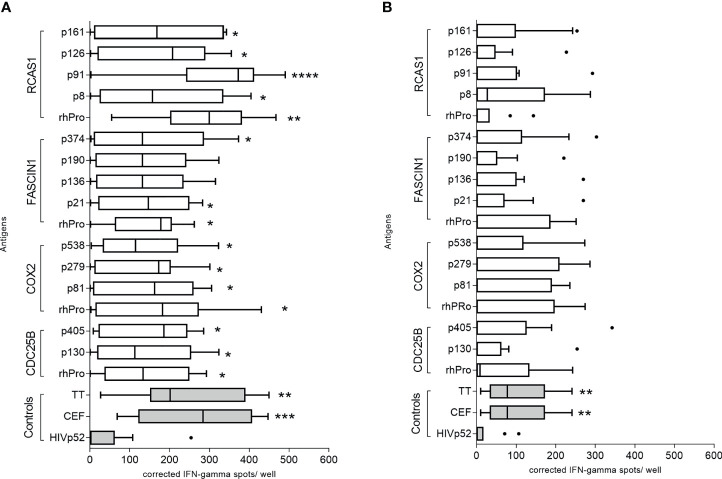
IFN-γ-secreting cells specific for both peptide and protein antigens can be identified in the peripheral blood of colorectal cancer patients. Corrected spots per well for **(A)** colorectal cancer patient or **(B)** volunteer donor PBMC stimulated with recombinant protein and individual peptides, presented as a box and whisker plots, horizontal line at median and Tukey outliers (filled circles). Negative control: HIVp52-66 and positive controls: pool of overlapping viral epitopes (CEF) and tetanus toxoid (TT). rhPro: recombinant human protein for that antigen set. *p < 0.05, ***p < 0.001, ****p < 0.0001 compared to the negative control. n = 10 colorectal cancer patients and 10 volunteer donors.

We evaluated 13 putative class II epitopes derived from CDC25B, COX2, FASCIN1, and RCAS1 and corresponding recombinant protein for IFN-γ secretion using PBMC derived from colorectal cancer patients and volunteer donors (**Supplementary Table 1**). Significant IFN-γ secretion was observed in colorectal cancer patients after stimulation with the majority of epitopes (**Figure 2A**). The CDC25B recombinant protein (mean, 138 ± 35 corrected spots per well (CSPW), p=0.042), and peptides CDC25B-p130 (median, 112 CSPW; range 10-278 CSPW, p=0.035) and CDC25B-p405 (mean, 152 ± 35 CSPW, p=0.021; **Figure 2A**) induced higher IFN-γ secretion as compared to an irrelevant peptide negative control (HIVp52). COX-2-derived epitopes p81 (mean, 150 ± 36 CSPW, p=0.026), p279 (mean, 135 ± 33 CSPW, p=0.040), and p538 (median, 114 CSPW; range 3-322 CSPW, p=0.019) as well as the recombinant protein (mean, 171 ± 44 CSPW, p=0.002; **Figure 2A**) induced a greater IFN-γ response than the negative control. Only recombinant FASCIN1 protein (mean, 144 ± 29 CSPW, p=0.018), FASCIN1-p21 (mean, 142 ± 35 CSPW, p=0.035) and FASCIN1-p374 (mean, 158 ± 44 CSPW, p=0.036; **Figure 2A**) induced significant IFN-γ secretion in colorectal cancer PBMC. IFN-γ secretion was significantly increased when PBMC were stimulated with the RCAS1 recombinant protein (mean, 277 ± 42 CSPW, p=0.005) and peptides RCAS1-p8 (mean, 184 ± 50 CSPW, p=0.022), RCAS1-p91 (median, 372; range, 5-458 CSPW, p<0.0001), RCAS1-p126 (mean, 172 ± 43 CSPW, p=0.019) and RCAS1-p161 (mean, 170 ± 46 CSPW, p=0.025; **Figure 2A**) as compared to the negative control.

There was no significant difference observed when the median response of the volunteer cohort for each peptide was compared to the median HIVp52 response, (p>0.05 for all epitopes and recombinant protein; **Figure 2B**). There were individual volunteer donors who demonstrated IFN-γ responses to some of the identified epitopes and proteins (**Figure 2B**) and those positive responses were no different from the responses observed in colorectal cancer patients (p>0.05 for all). Donor PBMC from volunteer donors found to demonstrate significant IFN-γ secretion to the antigens were used to generate peptide specific T-cell lines which also responded to the corresponding recombinant protein (**Supplementary Figure 2**).

### IFN-γ Inducing Peptide-Based Vaccines Derived From CDC25B and COX2 Inhibit the Growth of MC38 *In Vivo*

The T-cell epitopes were highly homologous between mouse and human (median 96% (range 78-100); **Supplementary Table 1**). We immunized C57BL6/J mice with the identified epitopes to determine whether we could generate peptide and protein specific T-cells in preparation for *in vivo* tumor challenge. Immunization induced a significant IFN-γ immune response for peptides derived from CDC25B, COX2, and RCAS1 and the corresponding protein (p<0.001 for all **Supplementary Figures 3A, B, D** respectively). However, neither the FASCIN1 peptide nor recombinant protein generated an IFN-γ response in FASCIN1 peptide-vaccinated mice, thus, FASCIN1 was not evaluated further for anti-tumor activity (**Supplementary Figure 3C**).

**Figure 3:**
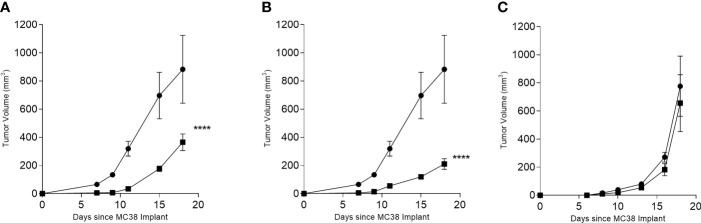
INF-γ-inducing peptide-based vaccines derived from CDC25B or COX2 inhibit the growth of MC38 *in vivo*. Mean (± SEM) tumor volume (mm^3^) in multi-peptide immunized mice (▪) or PBS immunized mice (●) for **(A)** CDC25B peptide mix, **(B)** COX2 peptide mix and **(C)** RCAS1 peptide mix. ****p < 0.0001, n = 4-8 mice/group.

C57BL6/J mice were immunized with multi-peptide vaccines derived from each antigen; CDC25B, COX2, and RCAS1. Only the CDC25B and COX2 significantly inhibited tumor growth. The mean tumor volume at the termination of the study from mice receiving the CDC25B (365 ± 165 mm^3^; **Figure 3A**) or COX2 multi-peptide vaccine (211 ± 106 mm^3^; **Figure 3B**) was significantly reduced as compared to the control (883 ± 481 mm^3^; p<0.001 for both). However, the mean tumor volume of mice receiving the RCAS1 multi-peptide vaccine (656 ± 189 mm^3^) was no different than the volume observed in control mice (775 ± 214 mm^3^; p=0.713; **Figure 3C**). Since the RCAS1 vaccine did not demonstrate anti-tumor activity the vaccine was not evaluated further in the experiments described below.

**Figure 4:**
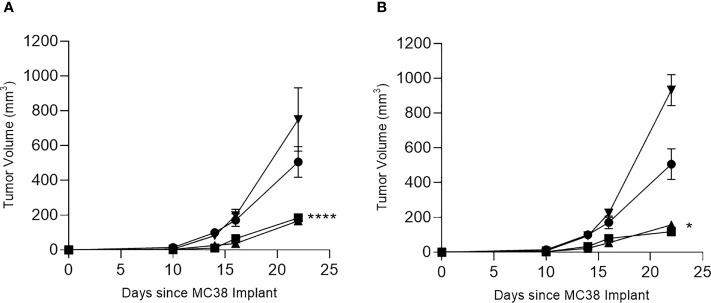
The anti-tumor efficacy of vaccination is dependent on CD8^+^ T cells. Mean (± SEM) tumor volume (mm^3^) from mice immunized with PBS (●) or with **(A)** CDC25B peptides or **(B)** COX2 peptides and treated with control IgG (▪), anti-CD8 (▼) or anti-CD4 (▲). *p < 0.05, ****p < 0.0001; n = 5 mice/group.

To determine which T-cell subset mediated the antitumor effect, we studied the *in vivo* response to the multi-peptide vaccines for COX2 and CDC25B in more detail. As expected, we observed significant MC38 tumor inhibition in mice immunized with the CDC25B (p<0.001; **Figure 4A**) or COX2 (p=0.043; **Figure 4B**) vaccine and treated with a control IgG. Treatment with anti-CD4 did not affect tumor inhibition and tumor volumes were similar to vaccine alone (p>0.49 for all). Depletion of CD8^+^ T-cells, however, abrogated the anti-tumor effect of either vaccine and tumor growth was no different from controls (p=0.858 for CDC25B and p=0.087 for COX2).

### Multi-Peptide Vaccination With Epitopes Derived From CDC25B or COX2 Prevents the Development of Tumors in Both the Colon And Small Intestine

**Figure 5:**
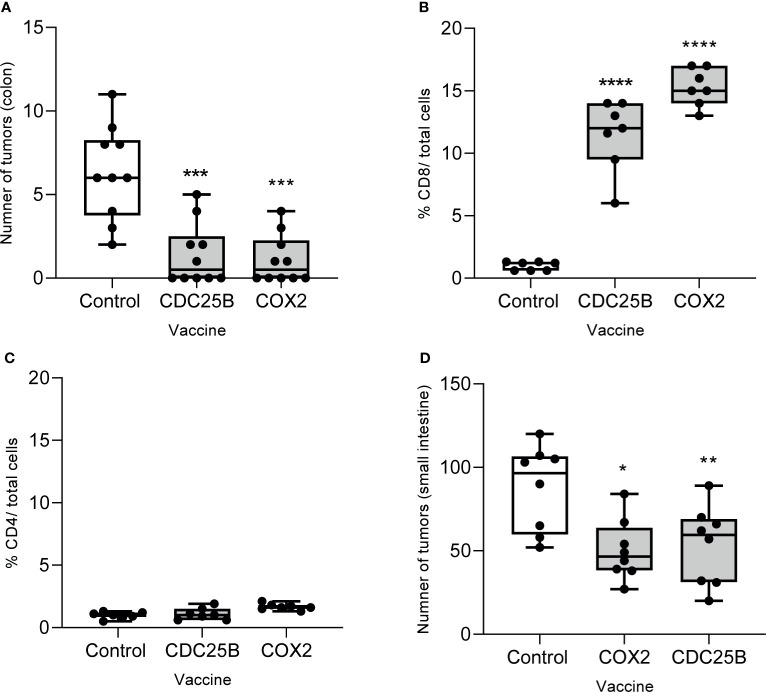
Multi-peptide vaccination with epitopes derived from CDC25B or COX2 prevents the development of tumors in both the colon and small intestine. **(A)** The number of tumors in the colon from AOM-treated mice immunized with the indicated vaccine; n = 10/group. Percent of **(B)** CD8^+^ or **(C)** CD4^+^ T cells out of total cells quantified by immunohistochemistry on tumors collected from AOM-treated mice after immunization with the indicated vaccine. n = 7/group. **(D)** Number of tumors in the small bowel after APC-min mice were immunized with the indicated vaccine; n = 8/group. All data are presented as box and whisker plots, horizontal line at median and whiskers min to max; *p < 0.05, **p < 0.01, ***p < 0.001, ****p < 0.0001.

We evaluated whether vaccination with epitopes from either CDC25B or COX2 could inhibit lesions in a spontaneous tumor model. As the vaccines had shown anti-tumor efficacy against MC38, we first evaluated efficacy in the AOM model. Mice immunized with CDC25B (mean, 1.1 ± 1.4 tumors) or COX2 peptides (mean, 1.4 ± 1.8 tumors) developed significantly fewer colon tumors as compared to the control (mean, 6.3 ± 2.8 tumors p<0.0001 for all; **Figure 5A**). Indeed, 50% of vaccinated mice in each group had no evidence of any lesions at the time of sacrifice.

We examined the phenotype of the tumor infiltrating T-cells in lesions that persisted after vaccination and observed that CD8^+^ T-cell levels were increased by 10-fold in tumors when the mice were immunized with the CDC25B peptide vaccine and by 16-fold after immunization with the COX2 peptide vaccine as compared to the control (p<0.001; **Figure 5B** and **Supplementary Figure 4**). The levels of CD4^+^ T-cells infiltrating the tumor were low and no different than levels observed in the control for mice immunized with CDC25B or COX2 peptides (p>0.55 for both; **Figure 5C** and **Supplementary Figure 4**).

We also examined whether the vaccine would inhibit the development of intestinal polyps in APC Min mice, which have a mutation in the APC gene. Mice immunized with CDC25B (mean, 53.4 ± 23.5 tumors) or COX2 peptides (50.3 ± 18.1 tumors) developed significantly fewer small bowel tumors as compared to control mice (mean, 87.5 ± 25.7 tumors; p<0.021 for all; **Figure 5D**).

## Discussion

Progress in the clinical translation of colon cancer vaccines would benefit from the identification of antigens suitable for immunization. Colorectal cancer is associated with a low mutation rate; therefore, neo-antigens do not play a major role in the immune modulation of most colorectal cancer subtypes (21). Many colorectal cancers associated immunogenic proteins are nonmutated and overexpressed in the tumor. Data presented here demonstrate that (1) patients with colorectal cancer can develop significant immune responses to nonmutated proteins that are important in driving the biology of the disease, (2) multi-epitope vaccines designed to elicit tumor specific CD4^+^ T-cells have potent anti-tumor activity, and (3) vaccines targeting colon cancer associated antigens can have prophylactic efficacy in spontaneous intestinal tumor models.

Epitopes predicted to bind human class II molecules could be identified for each of the antigens and the peptide specific T-cells generated recognize and respond to protein presented by autologous antigen presenting cells. Vaccines designed to stimulate tumor antigen specific T-cells, particularly those which elicit IFN-γ, uniquely modulate the tumor microenvironment. Tumor trafficking IFN-γ-secreting T-cells activate local antigen presenting cells and enhance cross-priming of tumor proteins resulting in a broadening of the immune response to additional antigens in the lesion (22). Cytokines, such as IFN- γ, promote the proliferation and recruitment of CD8^+^ T-cells into the tumor. IFN-γ can reverse functional defects in antigen presentation, including increasing upregulation of MHC I and allowing innate immune cells to more effectively present antigen to cytotoxic T-cells (23). The role of CD8^+^ T-cells in tumor eradication has been reported for other epitope-based IFN-γ-stimulating vaccines. The anti-tumor effect of peptide immunization targeting IGFBP-2 in mice challenged with a mammary cancer cell line was also dependent on CD8^+^ T-cells (15). These data underscore that vaccines designed to elicit IFN-γ-secreting T-cells have a broader immunologic impact than just increasing tumor-specific effector T-cells in the peripheral blood.

Both CDC25B and COX-2 can be expressed in high risk polyps that may progress to carcinomas, so we evaluated vaccination in two spontaneous tumor models for disease prophylaxis; AOM induced colon cancer and intestinal polyp formation in APC Min mice. Tumors caused by AOM are frequent in the distal part of the colon, resembling the location of spontaneous colorectal cancers in humans (24). The APC Min mouse has phenotypic and genetic similarities to human familial adenomatous polyposis although the numerous adenomas that develop in the animal are mainly located in the small intestine. The adaptive immune infiltrate in polyps consists of predominantly T-regulatory and Th17 cells (25). Immunization with either CDC25B or COX-2 epitopes could inhibit and even prevent tumor growth. Further, CD8^+^ tumor infiltrating T-cells were significantly induced with vaccination. In a recent meta-analysis of tumors derived from over 20,000 colorectal cancer patients, an increased number of CD8^+^ tumor infiltrating lymphocytes was associated with an improved cancer specific and overall survival (26). Vaccines, such as ours, that can elicit CD8^+^ tumor infiltrating lymphocytes could potentially have clinical benefit for the treatment and prevention of colon cancer.

One concern in immunizing non-cancer bearing individuals with vaccines targeting non-mutated proteins is the potential for the development of autoimmune disease. There are many reports of T-cells directed against non-mutated tumor antigens being identified in the peripheral blood of not only cancer patients, but also non-cancer bearing individuals. Both CD4^+^ and CD8^+^ T-cells specific for PRAME, a melanoma antigen, could be expanded from the peripheral blood of healthy donors (27). The expanded T-cell lines demonstrated anti-tumor activity *in vitro*. T-cells specific for multiple cyclin B1 specific CD4 T-cell epitopes could be expanded from both cancer patients and volunteer donors (28). One study, using tetramers directed against class II epitopes, identified T-cells specific for tyrosinase and NY-ESO in the peripheral blood of most healthy individuals evaluated (29). An investigation of multiple antigens in 114 blood donors found T-cells of both low and high avidity against WT-1 (15%), MUC1 (14%), PRAME (15%) and HER2 (5%) (30). Investigators hypothesized that the presence of these cells could be related to gonadal-testis expression and pregnancy. Several elegant studies have shown that clonal deletion in the thymus may eliminate some self-reactive T-cells, but many more remain in the periphery and are under the control of peripheral tolerance (31). These self-epitopes were not presented by thymic antigen presenting cells. Indeed, investigations have shown that self-peptide specific T-cells in the blood of healthy individuals are found at frequencies similar to non-self-peptides (32). Presumably, this large self-reactive T-cell pool would prevent holes in the T-cell repertoire that pathogens could exploit (32). The large self-reactive T-cell pool provides a memory population to exploit for cancer vaccines, albeit mechanisms of peripheral tolerance must be overcome.

There is a long history of immunizing cancer patients, in the therapeutic setting, with vaccines directed against such antigens. Investigators evaluated the toxicity profile of vaccines tested in over 200 phase I clinical trials. In the 4,942 patients assessed, the rate of greater than Grade 3 adverse events was 1.25 events per 100 patients (1). While the low adverse event rate associated with vaccines targeting non-mutated cancer related proteins is encouraging, as studies move into the prophylactic setting close monitoring for autoimmune symptomology must be considered.

Colorectal cancer is one of the most common solid tumors impacting patients and even those with early stage disease have a risk of relapse after optimal treatment. Vaccines directed against proteins maintaining the malignant phenotype have the potential to limit recurrence or even the development of disease. Studies in pre-clinical models have shown that multiple antigen cancer vaccines are more clinically effective than immunizing with a single antigens alone (3). Work described here provides proof of principle that multiple nonmutated tumor antigens can be identified for use in a vaccine for colorectal cancer therapy and prevention.

## Data Availability Statement

The raw data supporting the conclusions of this article will be made available by the authors, without undue reservation.

## Ethics Statement

The studies involving human participants were reviewed and approved by University of Washington Human Subjects Division. The patients/participants provided their written informed consent to participate in this study. The animal study was reviewed and approved by University of Washington Animal Care and Use Committee.

## Author Contributions

MD and RL contributed to conception and design of the study. LC, EG, and MK performed the *in vivo* and *in vitro* experiments. LC performed the statistical analyses. DC wrote the first draft of the manuscript. All authors contributed to the article and approved the submitted version.

## Funding

NCI contract HHSN261200433001C and the Fundación Logrand. MLD is supported by the Helen B. Slonaker Endowed Professor for Cancer Research and an American Cancer Society Clinical Professorship (CRP-15-106-01-LIB).

## Conflict of Interest

MD is a stockholder in EpiThany and receives grant support from Celgene, EMD Serono, Pfizer, Janssen, and Precigen.

The remaining authors declare that the research was conducted in the absence of any commercial or financial relationships that could be construed as a potential conflict of interest.

## Publisher’s Note

All claims expressed in this article are solely those of the authors and do not necessarily represent those of their affiliated organizations, or those of the publisher, the editors and the reviewers. Any product that may be evaluated in this article, or claim that may be made by its manufacturer, is not guaranteed or endorsed by the publisher.

## Supplementary Material

Supplementary Table 1 Predicted MHC Class II binding epitopes. Orange highlighted epitopes were validated as responding to protein.

Supplementary Figure 1 Example Western blot validation of IgG antibody response to colorectal cancer antigens. **(A)** CDC25B; 62kD **(B)** COX2; 115kD **(C)** FASCIN1; 60 kD and **(D)** RCAS1; 50kD. (l) polyclonal antibody specific for the antigen, (ll) a representative ELISA positive patient sample, (lll) an ELISA negative subject sample. Molecular weight protein marker (far left).

Supplementary Figure 2 Epitope-specific cells respond to naturally processed protein. IFN-γ ELISPOT cSPW (corrected spots per well) for **(A)** CDC25B-p130-specific T-cells, **(B)** CDC25B-p405specific T-cells, **(C)** COX2-p81-specific T-cells, **(D)** COX2-p279-specific T-cells, **(E)** COX2-p538-specific T-cells, **(F)** Fascin-p384 specific T-cells, **(G)** RCAS1-p8-specific T-cells and **(H)** RCAS1-p91-specific T-cells stimulated with the expansion peptide or corresponding recombinant protein. TRIP13-p141 and recombinant Cyclin B are used as negative peptide and protein controls, respectively; ****p < 0.0001, ***p < 0.001, **p < 0.01, *p < 0.05.

Supplementary Figure 3 CDC25B, COX2, RCAS1, but not FASCIN1 peptides and protein elicit an IFN-Ɣ response in mice. Mean (±SEM) IFN-γ corrected spots per well (cSPW) when splenocytes were stimulated with the indicated antigen after vaccination with peptides derived from **(A)** CDC25B, **(B)** COX2 **(C)** FASCIN1, and **(D)** RCAS1. *p < 0.05, ***p < 0.001 as compared to the negative control HIVp52 peptide.

Supplementary Figure 4 Representative immunohistochemistry for CD8 and CD4 on tumors collected from the colon after immunization with the indicated vaccine. Positive T-cells are stained green and all cells are stained blue.

## Abbreviations

AOM, Azoxymethane; cSPW, Corrected spots per well; IFN-γ, Interferon-gamma; PBMC, Peripheral blood mononuclear cells; SPW, Spots per well; SEM, Standard error of measurement.
