# Supplementary material for: Multi-Epitope-Based Vaccines for Colon Cancer Treatment and Prevention
Source: Front Immunol. 2021 Aug 30;12:729809. doi: 10.3389/fimmu.2021.729809 (PMC8437302; doi:10.3389/fimmu.2021.729809)
Supplement: Supplementary Table 1 — Predicted MHC Class II binding epitopes. Orange highlighted epitopes were validated as responding to protein. [file Presentation_1.pdf]

SUPPLEMENTAL TABLES AND FIGURES

Supplemental Table 1. Predicted MHC Class II binding epitopes

Protein	number	# aa	sequence	% Homology of peptide with mouse
CDC25B	p130-150	21	qaiqaasriirneqfairrfq	78%
	p405-427	23	vdgkhqdlkyispetmvalltgk	100%
COX2	p81-96	16	fkgfwnvvnnipflrn	87%
	p279-295	17	glvpglmmyatiwlreh	100%
	p538-553	16	gevgfqiintasiqslic	94%
FASCIN1	p374-p398	25	elflmklinrpiivfrgehgfigcr	100%
	p136-p154	19	iamhpqvniysvtrkryah	100%
	p21-p40	20	nkyltaeafgfkvnasassl	100%
	p190-p209	20	tadhrflrhdgrlvarpepa	95%
RCAS1	p8-p27	20	lfkfctclatvfsflkrlic	95%
	p91-p110	20	epdyfkdmtptirktqkivi	100%
	p126-148	23	gfssrlaatqdlpfihqsselgd	96%
	p161-181	21	eeedaawqaeevlrqqkladr	95%

Orange highlighted epitopes were validated as responding to protein.

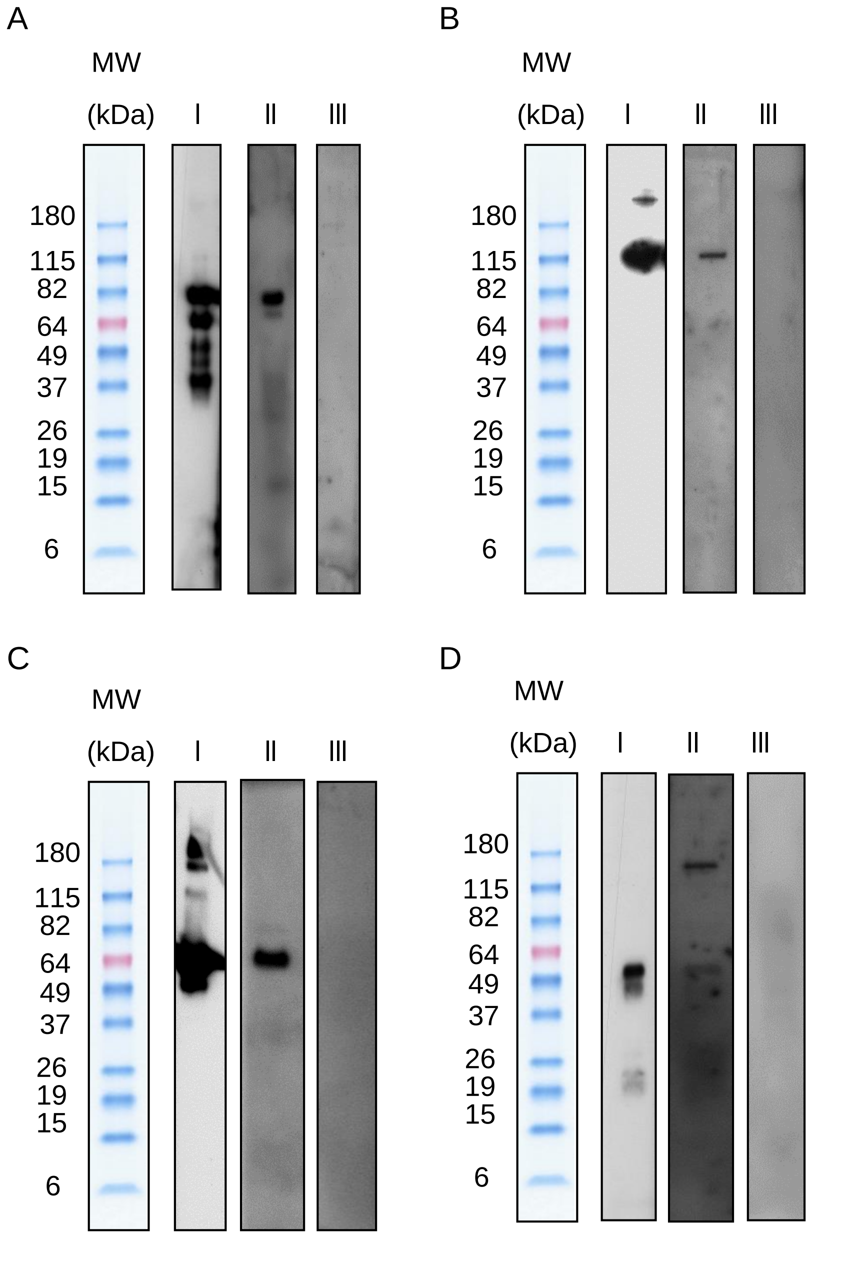

Supplemental Figure 1. Example Western blot validation of IgG antibody response to colorectal cancer antigens. (A) CDC25B; 62kD (B) COX2; 115kD (C) FASCIN1; 60 kD and (D) RCAS1; 50kD. (I) polyclonal antibody specific for the antigen, (II) a representative ELISA positive patient sample, (III) an ELISA negative subject sample. Molecular weight protein marker (far left).

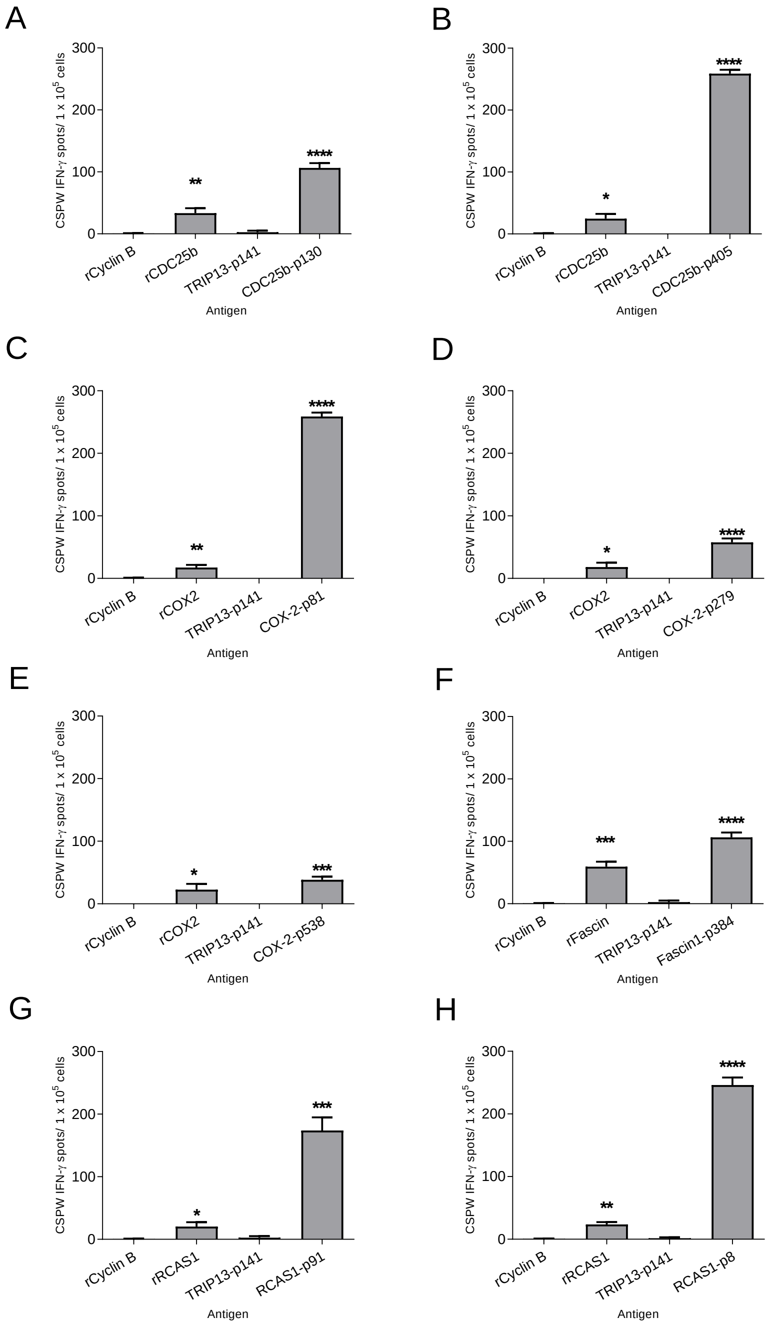

Supplemental Figure 2. Epitope-specific Th1 respond to naturally processed protein. IFN-γ ELISPOT cSPW (corrected spots per well) for (A) CDC25B-p130-specific T-cells, (B) CDC25B-p405specific T-cells, (C) COX2-p81-specific T-cells, (D) COX2-p279-specific T-cells, (E) COX2-p538-specific T-cells, (F) Fascin-p384 specific T-cells, (G) RCAS1-p91-specific T-cells and (H) RCAS1-p8-specific T-cells stimulated with the expansion peptide or corresponding recombinant protein. TRIP13-p141 and recombinant Cyclin B are used as negative peptide and protein controls, respectively; ****$p<0.0001$, ***$p<0.001$, **$p<0.01$, *$p<0.05$.

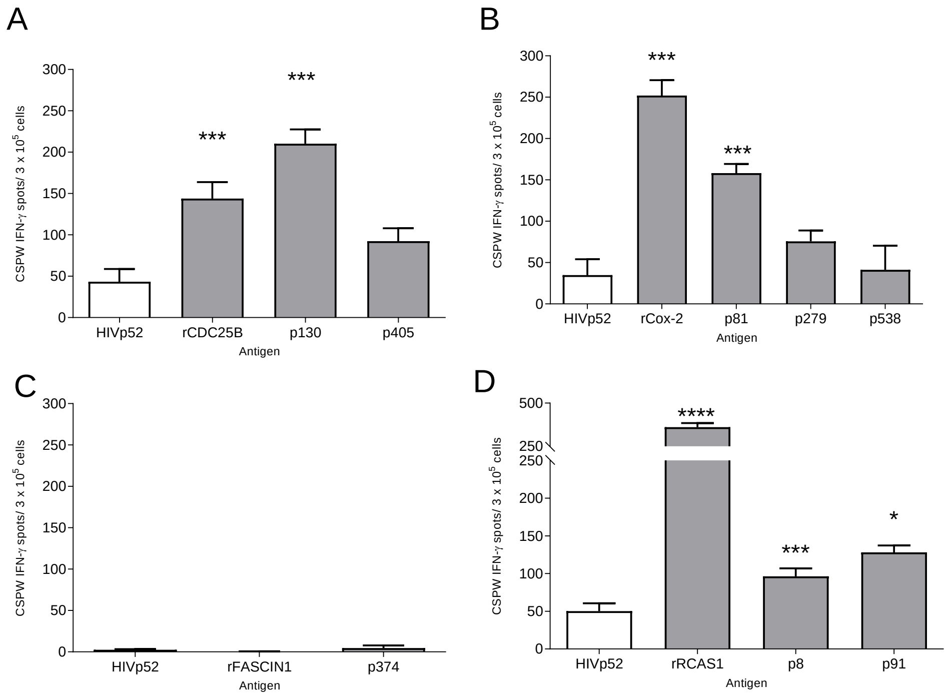

Supplemental Figure 3. CDC25B, COX2, RCAS1, but not FASCIN1 peptides and protein elicit an Th1 response in mice. Mean (±SEM) IFN-γ corrected spots per well (CSPW) when splenocytes were stimulated with the indicated antigen after vaccination with peptides derived from (A) CDC25B, (B) COX2 (C) FASCIN1, and (D) RCAS1. $*p<0.05$, $***p<0.001$, $****p<0.0001$ as compared to the negative control HIVp52 peptide.

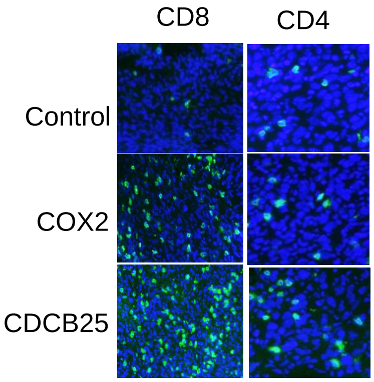

Supplemental Figure 4. Representative immunohistochemistry for CD8 and CD4 on tumors collected from the colon after immunization with the indicated vaccine. Positive T-cells are stained green and all cells are stained blue.
